# Rigosertib-Activated JNK1/2 Eliminate Tumor Cells through p66Shc Activation

**DOI:** 10.3390/biology9050099

**Published:** 2020-05-15

**Authors:** Julia K. Günther, Aleksandar Nikolajevic, Susanne Ebner, Jakob Troppmair, Sana Khalid

**Affiliations:** 1 Daniel Swarovski Research Laboratory (DSL), Department of Visceral, Transplant and Thoracic Surgery (VTT), Medical University Innsbruck (MUI), 6020 Innsbruck, Austria; julia.guenther@i-med.ac.at (J.K.G.); aleksandar.nikolajevic@i-med.ac.at (A.N.); susanne.ebner@i-med.ac.at (S.E.); 2 Department of Oral Biology, School of Dental Medicine, University of Pittsburgh, Pittsburgh, PA 15213, USA

**Keywords:** p66Shc, Rigosertib, reactive oxygen species, cell death

## Abstract

Rigosertib, via reactive oxygen species (ROS), stimulates cJun N-terminal kinases 1/2 (JNK1/2), which inactivate RAS/RAF signaling and thereby inhibit growth and survival of tumor cells. JNK1/2 are not only regulated by ROS—they in turn can also control ROS production. The prooxidant and cell death function of p66Shc requires phosphorylation by JNK1/2. Here, we provide evidence that establishes p66Shc, an oxidoreductase, as a JNK1/2 effector downstream of Rigosertib-induced ROS production, DNA damage, and cell death. This may provide a common pathway for suppression of tumor cell growth by Rigosertib.

## 1. Introduction

Mutational activation of the three small G proteins HRAS, KRAS and NRAS is common in tumors, enabling them to interact with various effectors—among which the members of the serine-threonine kinases of the RAF family (ARAF, BRAF, CRAF), PI3K, and RalGDS feature prominently [1,2,3]. RAS has long been a target for possible therapeutic intervention, however, it is still considered for the most part “undruggable” [3]. The focus thus has been on downstream targets, and inhibitors for mutant BRAF kinase and MEK have entered the clinic [3,4]. The development of small molecules, which interfere with RAS-binding to downstream effectors, is also extensively pursued. Rigosertib (also known as ON01910 and Estybon) is a synthetic benzyl styryl sulfone, currently in clinical trial for high-risk myeloid dysplastic syndrome [5]. Originally developed as a non-ATP competitive multi-kinase inhibitor, the precise kinase target(s) of Rigosertib remains elusive. An alternative mechanism of Rigosertib action has been described whereby it interacts with the RAS-binding domains (RBDs) of RAS effectors. This interferes with signal flow from oncogenic RAS [6]. However, more recent findings dispute this notion and provide a different explanation for the inhibition of signaling downstream of RAS and RAF [7]. Rigosertib activates cJun N-terminal kinases 1/2 (JNK1/2) that, in turn, phosphorylate the guanine nucleotide exchange factor (GEF) SOS and the RAF kinases CRAF and BRAF. This modification prevents RAS/RAF downstream signaling with negative effects on cell survival and proliferation. JNK kinases also have alternative means to induce cell death. JNK signaling caused by Rigosertib contributes to cell death in chronic lymphatic leukemia (CLL) through the production of ROS [8]. JNK kinases can also directly translocate to mitochondria under different stress conditions and cause ROS production and cell death [9,10]. We recently tied JNK1/2 to the activation of p66Shc [11], which belongs to the SHCA proteins. This family consists of three isoforms termed p66Shc, p52Shc, and p46Shc, according to their molecular weights [12]. The two smaller isoforms function as adapter proteins in the transmission of mitogen signaling to RAS [12], while p66Shc has been linked to mitochondrial ROS production [13]. The p66Shc activation requires phosphorylation of S36 by JNK1/2, followed by additional phosphorylations on S139, T206, and S213 by PKCβ [11,14]. This process is responsible for the subsequent translocation of cytosolic p66Shc to the mitochondria, where it causes the production of ROS [13]. In all cases studied, ROS were responsible for cell death and functional impairment in many diseases and pathological conditions [12]. Since Rigosertib activates JNK1/2 [7], we are interested in a possible p66Shc requirement for tumor-cell killing after Rigosertib treatment.

## 2. Material and Methods

### 2.1. Cell Culture and Cell Analysis

The MCF7 cell line was obtained from LGC Standards (Wesel, Germany) for these experiments, and cells were maintained in Minimal Essential Medium (MEM) (PAA Laboratories, Pasching, Austria). PC3 and DU-145 cells were provided by Prof. Helmut Klocker, University Hospital for Urology, Division of Experimental Urology, Department of Surgery, Medical University Innsbruck, Austria, and cells have been authenticated by short tandem repeat analysis using the AmpFlSTR^®^ SGM Plus^®^ PCR amplification kit (Applied Biosystems, Foster City, CA, USA) at the Institute of Legal Medicine, Medical University of Innsbruck, Innsbruck, Austria. PC3 and DU-145 cells were kept in RPMI1640 medium. All media contained 10% fetal calf serum (FCS), 2 mmol/L l-glutamine, and 1× penicillin/streptomycin (all from PAA Laboratories, Pasching, Austria). Cells were maintained at 37 °C in a 5% CO_2_/95% air-humidified atmosphere. The cells were passaged every alternate day. For drug treatment, 350,000 cells were seeded per well of a 6-well plate and grown for 24 h to obtain 70–80% confluence. The medium was replaced with 0.1% FCS containing medium [7] along with 50 µM Rigosertib (Selleckchem, Munich, Germany), while the same volume of the solvent DMSO was added to control wells. The JNK1/2 inhibitor SP600125 (LC Laboratories, Woburn, MA, USA) was supplied at a final concentration of 20 µM one h prior to Rigosertib.

### 2.2. Protein Work and Antibodies

Proteins were isolated and detected as described previously [11,14,15]. Primary antibodies raised against the following proteins were used: phospho-γH2AX (#2577); phospho-cJun (#9261); PARP (#9542); ERK1/2 (#4695) from Cell Signaling Technology, Boston, MA, USA; phospho-ERK1/2 (sc-136521); JNK (sc-571) from Santa Cruz Biotechnology, Santa Cruz, CA; α-tubulin (T5168, Sigma Aldrich, Dorset, UK); Shc1 (610879, BD Biosciences, San Diego, CA, USA); pSer36-Shc1 (54518, Abcam, Cambridge, UK); GAPDH (#AM4300, Invitrogen, Eugene, OR, USA); and anti-HA-Peroxidase (12013819001, Roche, Mannheim, Germany). Proteins were visualized by ECL Western blot detection reagent (Amersham, Buckinghamshire, UK), and quantified by densitometric scanning and image analysis via ImageJ (NIH, Bethesda, MD, USA).

### 2.3. Cell Death Detection

Cell viability was assessed using AnnexinV/PI staining, as previously described [11]. DNA damage and cell death were also monitored by antibodies detecting phosphorylation of γH2AX and cleavage of PARP, respectively.

### 2.4. p66Shc Plasmid Transfection

MCF7 cells were transfected with 1 µg pRec_p66Shc-HA-His (GeneCopoeia, Rockville, MD, USA) using Lipofectamine 2000 (Invitrogen, Eugene, OR, USA). Transiently transfected cells were used 48 h after transfection.

### 2.5. Statistics

All data are presented as a mean  ±  SD following *t*-test or ANOVA analyses. Statistical analyses were done using GraphPad Prism 5 (GraphPad Software, La Jolla, CA, USA). Significance values were designated as follows: * *p*  <  0.05, ** *p*  <  0.005, and *** *p*  <  0.0005.

## 3. Results

### 3.1. Effect of Rigosertib Treatment on JNK1/2 and ERK1/2 Activity in Tumor Cells

**Figure 1:**
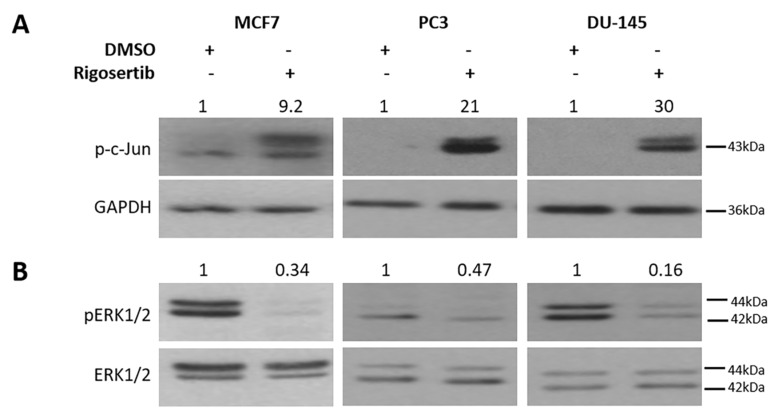
Effect of Rigosertib on MAPK signaling. Representative Western blots show an increase in cJun N-terminal kinases 1/2 (JNK1/2) (**A**) and a decrease in ERK1/2 activity (**B**) in MCF7, PC3, and DU-145 cells stimulated with 50 μM Rigosertib for 18 h. Numbers on top of the blot indicate the fold change in protein phosphorylation upon Rigosertib treatment (normalized to loading control) relative to DMSO (solvent)-treated control samples. All experiments have been repeated at least three times with consistent results. A representative blot is shown.

Three different cancer cell lines were included in our studies: the breast cancer line MCF7 and the prostate cancer lines PC3 and DU-145. JNK1/2 activity was monitored by analyzing S63 phosphorylation of cJun [16]. Pronounced activation occurred in all cell lines following treatment with 50 µM Rigosertib for 18 h. As observed before [7], the activation of ERK1/2 was significantly reduced by Rigosertib in MCF7 cells. A similar response was observed in DU-145 and PC3 cells (Figure 1). These data confirm the previously reported opposing effect of Rigosertib on the activation of the mitogen activated protein kinases (MAPKs) cJun N-terminal kinases (JNK1/2) and extracellular signal-regulated kinases ERK1/2 [7].

### 3.2. Rigosertib Treatment Activates p66Shc and Causes Cell Damage

**Figure 2:**
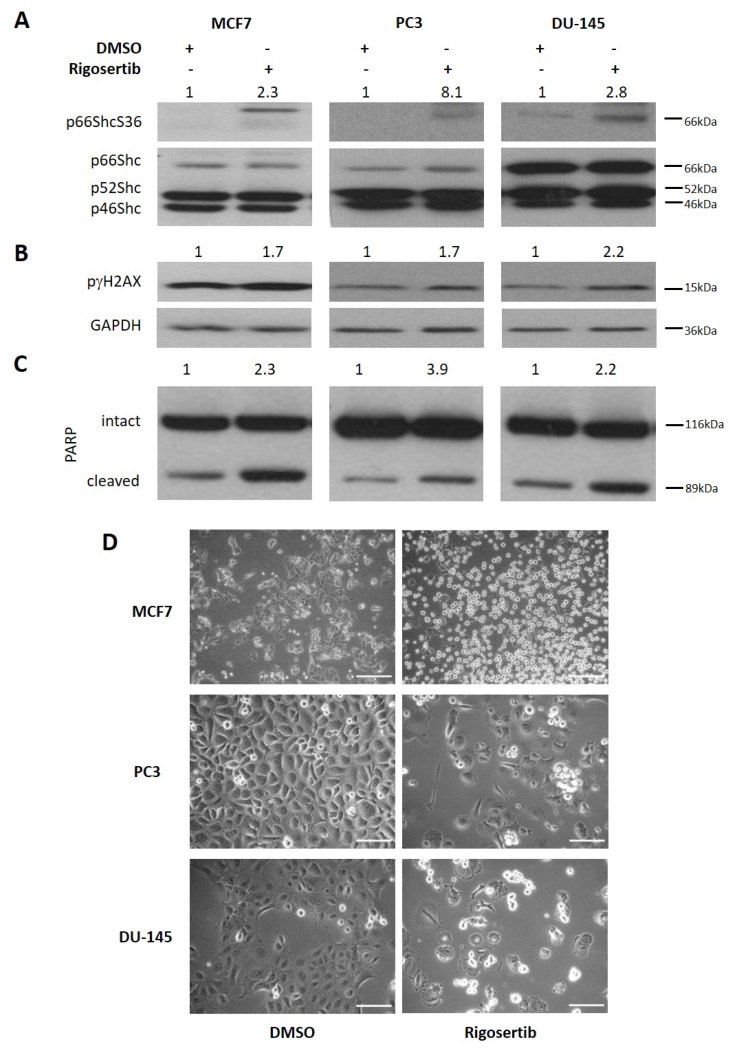

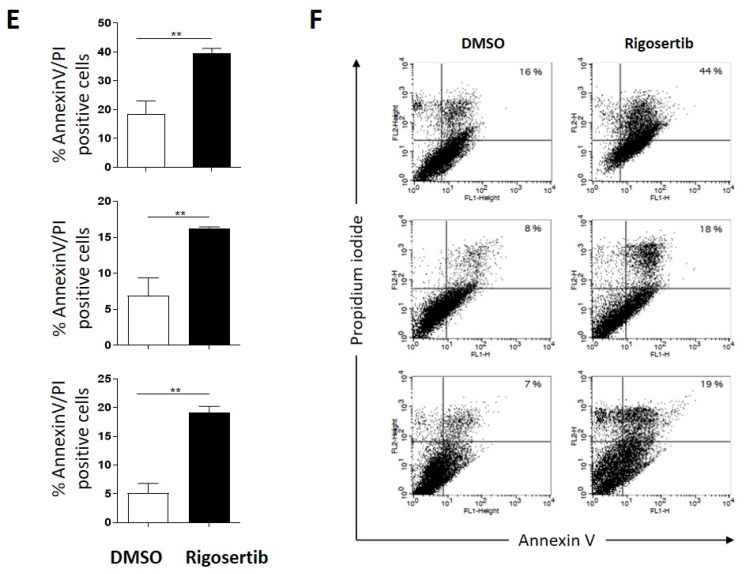
Rigosertib increases p66Shc activity and cell death in tumor cell lines. Representative Western blots show an increase in p66Shc activity (**A**), γH2AX phosphorylation (**B**), and PARP cleavage (**C**) in MCF-7, PC3, and DU-145 cells stimulated with 50 μM Rigosertib for 18 h. Numbers on top of the blot indicate the fold change in protein phosphorylation upon Rigosertib treatment (normalized to loading control) relative to DMSO (solvent)-treated control samples. For PARP, the ratio of cleaved and intact protein is shown. MCF-7, PC3, and DU-145 were imaged in phase contrast to detect cellular morphology (**D**) and analyzed for cell death by Annexin/PI after treating cells with either Rigosertib (50 μM) or DMSO for 96 h. Results are presented as % of Annexin V-positive and PI-positive cells (**E**) and scattered plots (**F**). The drug-containing medium was refreshed after 48 h during 96 h incubation time. All experiments have been repeated at least three times with consistent results, except the Annexin/PI analysis for DU-145, which has been repeated twice. Values shown are mean ± S.D. A representative blot is shown. Scale bar size: 100 µm.

We next addressed whether JNK1/2 activation results in the phosphorylation of S36 on p66Shc, an event that is essential for the activation of its prooxidant and pro-death activity [12]. As shown in Figure 2A, levels of unphosphorylated p66Shc protein differed among the cell lines studied with DU-145 showing the highest p66Shc expression. No such pronounced differences were observed with the smaller isoforms p46Shc and p52Shc. An increase in p66ShcS36 phosphorylation was evident in all three cell lines tested (Figure 2A). As shown in Figure 2B, this went along with enhanced phosphorylation of the DNA damage marker γH2AX and increased cleavage of PARP, suggesting apoptosis (Figure 2C). Cell death was also evident from the microscopic imaging of cell monolayers, which showed detachment of cells (Figure 2D), and from the increase in the number of AnnexinV/PI positive cells (Figure 2E,F). These data suggest that following Rigosertib treatment, activation of p66Shc occurs, which in many published studies has been shown to be essential for cell death induction [12].

### 3.3. p66Shc Activation Requires JNK1/2 Activity

**Figure 3:**
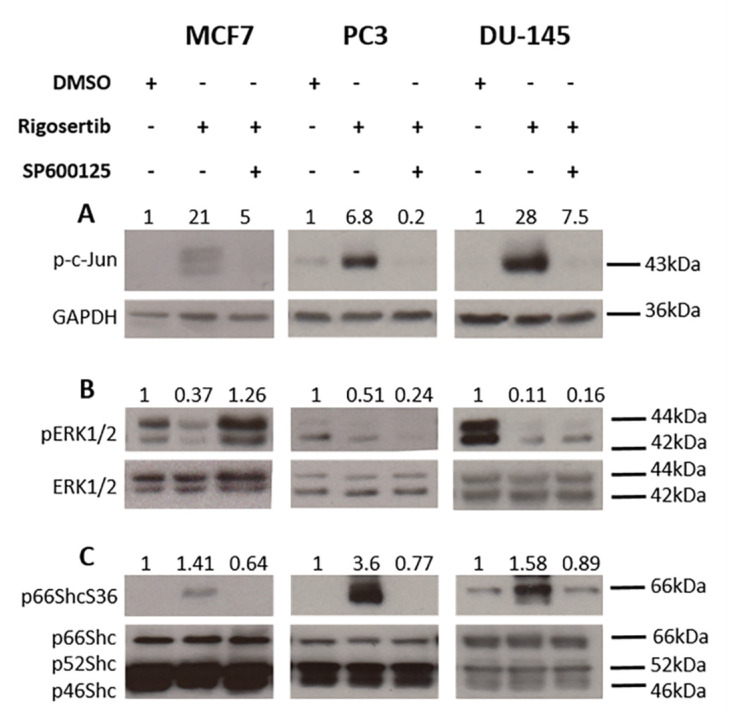
JNK1/2 regulation of p66Shc activity in tumor cell lines upon Rigosertib treatment. Representative Western blots demonstrating the effects of SP600125 (20 μM, JNK inhibitor) treatment before Rigosertib application (50 μM for 18 h) on cJun (**A**), ERK1/2 (**B**), and p66ShcS36 phosphorylation (**C**) in MCF7, PC3, and DU-145 cells. Numbers on top of the blot indicate the fold change in protein phosphorylation upon Rigosertib treatment (normalized to loading control) relative to DMSO (solvent)-treated control samples. All experiments have been repeated at least three times with consistent results. A representative blot is shown.

To confirm the involvement of JNK1/2 in the activation of p66Shc, cells were pretreated with the JNK1/2 inhibitor SP600125 for one h at a concentration of 20 µM prior to Rigosertib treatment. As expected, SP600125 efficiently prevented cJun phosphorylation, which we monitored to assess JNK1/2 activity (Figure 3A). In MCF7 cells, the presence of the inhibitor restored ERK1/2 activation in Rigosertib-treated cells (Figure 3B), while no similar effect was observed in PC3 and DU-145 cells. These findings suggest that in contrast to a previous report [7], suppression of ERK1/2 signaling can be achieved in a JNK1/2-independent fashion. However, Rigosertib-induced p66ShcS36 phosphorylation was completely blocked by inhibition of JNK1/2 (Figure 3C).

### 3.4. JNK1/2 and p66Shc Are Required for Cell Damage

**Figure 4:**
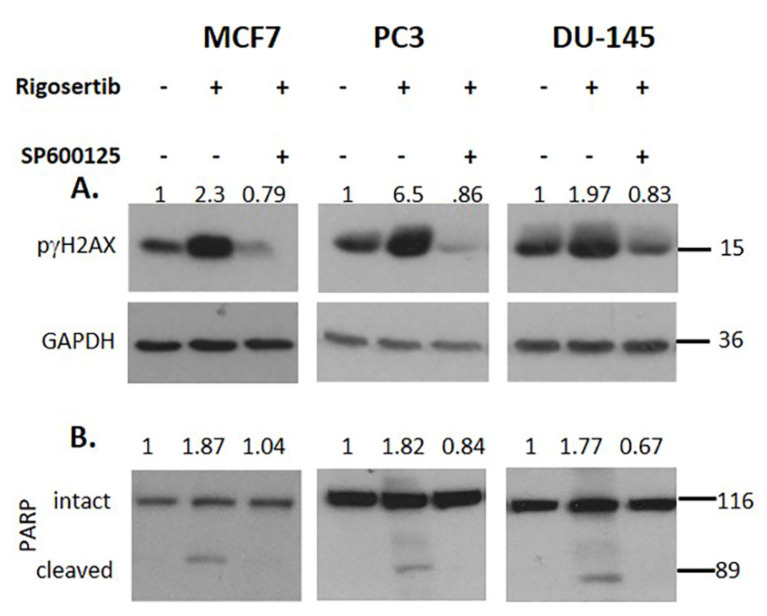
Rigosertib-mediated tumor cell killing is reduced by JNK1/2 inhibition. Representative Western blots documenting the effect of SP600125 treatment (20 μM, added one h before Rigosertib) on γH2AX phosphorylation (**A**) and PARP cleavage (**B**) in MCF7, PC3 and DU-145 cells. Rigosertib was applied for 18 h at a concentration of 50 µM). Numbers on top of the blot indicate the fold change in protein phosphorylation upon Rigosertib treatment (normalized to loading control) relative to DMSO (solvent) treated control samples. All experiments have been repeated at least three times with consistent results. A representative blot is shown.

**Figure 5:**
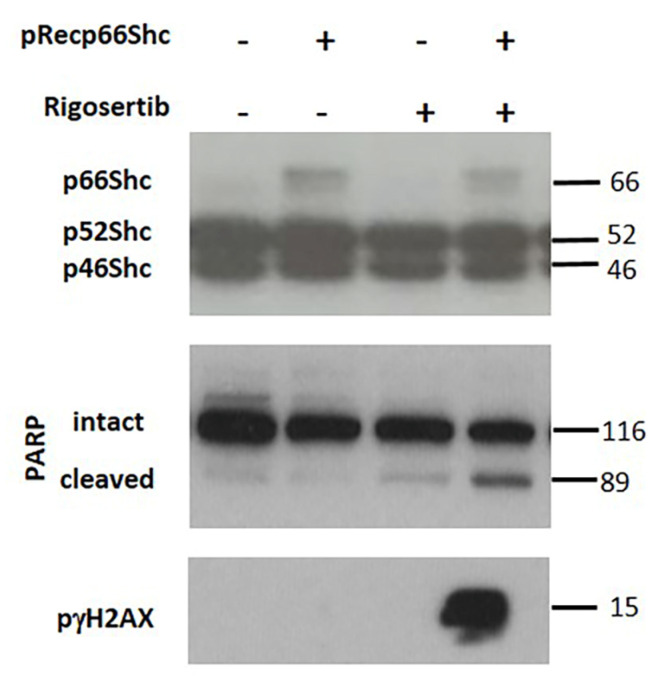
During the submission of the revised manuscrip an incorrect version of this Figure has been submitted, The figure inserted now is as requested by the reviewers. p66Shc is necessary for Rigosertib-mediated tumor cell killing. Representative Western blots demonstrate γH2AX phosphorylation and PARP cleavage in p66Shc-HA-His transiently transfected MCF7 cells treated with Rigosertib (50 μM) or DMSO for 18 h. All experiments have been repeated at least three times with consistent results. A representative blot is shown.

Treatment with the JNK inhibitor SP600125 prior to Rigosertib prevented the phosphorylation of γH2AX (Figure 4A) as well as the cleavage of the caspase substrate PARP (Figure 4B) in all three cell lines.” These data demonstrate the involvement of JNK1/2 in these processes. To further corroborate that p66Shc is required for cellular damage, we overexpressed p66Shc in MCF7 cells. Elevated p66Shc levels dramatically increased γH2AX phosphorylation and PARP cleavage (Figure 5).

## 4. Discussion

Rigosertib was discovered in a phenotypic screen for molecules that induce mitotic cell-cycle arrest in tumor cells while sparing non-transformed cells [17]. The direct target of Rigosertib remains elusive, and inhibition of PLK1 [17] and PI3K [18] have been reported. Antitumor activities also involve the induction of oxidative stress [8], or a possible function as a RAS mimetic, which disrupts RAS binding to downstream effectors to inhibit signaling [6]. Most recently, Ritt et al. provided evidence that the effect of Rigosertib on RAS/RAF signaling may not be direct but that Rigosertib causes JNK1/2 activation resulting in the inhibitory phosphorylation of SOS, CRAF, and BRAF, and prevention of downstream survival and proliferation signaling [7]. JNK1/2 activation requires induction of mitochondrial ROS production by Rigosertib [7].

**Figure 6:**
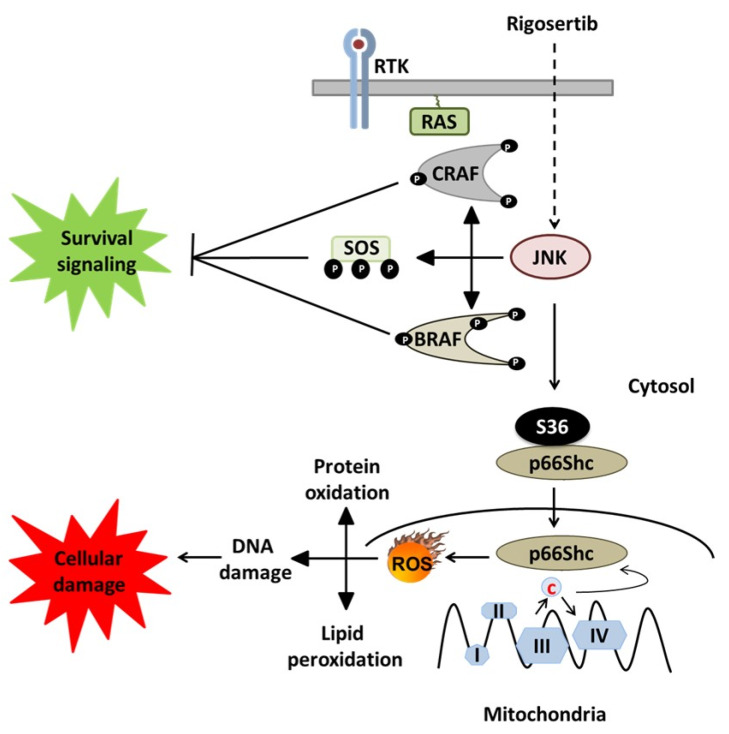
Rigosertib-mediated cell death pathways (modified from Ritt et al. [7]). Work by Ritt et al. identified the effect of Rigosertib-induced JNK activation on the suppression of the RAS signaling pathway and consequent blunting survival and growth signals. Our data point to an additional mechanism, which may be even more common, linking activated JNK1/2 to the activation of p66Shc, which has been implicated in ROS-mediated cell death under various conditions [12].

In the work presented here, we confirm earlier findings on Rigosertib-induced JNK1/2 activation and concomitant inhibition of ERK signaling in MCF7 cells [7] and show a similar effect for PC3 and DU-145 cells. However, JNK1/2 activation was not responsible for ERK1/2 inhibition in the latter two cell lines. This implies the existence of additional mechanisms through which JNK1/2 can compromise cell survival, besides the previously discovered phosphorylation-inactivation of key RAS pathway components causing inhibition of ERK1/2 activation [7]. In our experiments, we describe an additional JNK effector pathway, which may affect cell survival via the activation of p66Shc (Figure 6).

JNK1/2 are also required for the activation of p66Shc [11], which is essential for mitochondrial ROS production and cell death induction in various pathological settings [12]. We therefore have tested whether the JNK1/2-p66Shc axis provides an alternative pathway to Rigosertib-induced cell killing. Here, we demonstrate that JNK1/2 activation by Rigosertib results in the phosphorylation of S36 on p66Shc. Previously published work has confirmed that this is an essential step in the activation of the prooxidant and pro-death activity of p66Shc [13]. While we have not directly measured ROS levels in our cells, the work by Ritt et al. already confirmed Rigosertib-induced ROS production. They also confirmed that it was essential for the inhibition of ERK signaling [7]. Published work consistently confirmed the requirement of ROS for the biological effects of p66Shc [12,13]. p66Shc has been extensively studied in conditions where excessive ROS production contributes to pathologies ranging from ischemia-reperfusion injury, obesity, and diabetes to neurodegenerative disorders [12]. Regarding cancer, the role of ROS is more complex. High ROS levels have been reported for many tumors. However, tumors also remain sensitive to a further increase in ROS production. Indeed, this may also be a mechanism through which many cancer therapeutics kill cancer cells [19]. Thus, ROS, and by implication ROS producing systems, can have oncogenic and tumor suppressive functions. p66Shc is overexpressed in various tumors, which display elevated ROS levels [12]. Moreover, p66Shc overexpression in MCF7 cells resulted in cell killing [20]. Treatment of prostate cancer cells with the naturally occurring compound phenethyl isothiocyanate (PEITC) resulted in ROS-dependent cell death, which required p66Shc [21]. Increasing ROS production via p66Shc may thus be of therapeutic interest. Our results suggest that JNK1/2 activation following Rigosertib treatment cannot only block tumor cell growth via the inhibition of RAS/RAF/MEK/ERK signaling, but also additionally through unleashing p66Shc-dependent ROS stress.

## Data Availability

The datasets supporting the conclusions of this article are included within the article.
